# The Greatest Medical Library of the World

**Published:** 1890-11-01

**Authors:** 


### The Greatest Medical Library of the World.

The largest collection of medical literature in the world must always be a matter of interest to physicians, if only as a matter of curiosity as to it size and location, while to medical investigators and writers it is often of considerable practical importance toknowwhere they are most likely to find the information which they need to complete their work. At the present time this collection exists in Washington, the capital of the United States, as the, so called, Library of the Surgeon-General's Office of the United States Army, and, while its existence is probably well-known to most of our readers, it may be that comparatively few of them are familiar with its history, extent, or practical utility. At this time it contains about 95,000 volumes and 140,000 pamphlets, using the word "volumes" in the conventional sense as applying to books of 100 pages or more, while pamphlets refers to those of smaller size.

The total number of distinct medical works which have been printed since the invention of movable types is unknown, but if we include the various editions in which there is any substantial difference, it has been estimated that one copy of each would make about 140,000 volumes and 250,000 pamphlets, on which basis of calculation the Washington library contains about two thirds of the whole.

We cannot, however, judge fairly of a library from its size alone, quality as well as quantity must be considered. The Washington collection is rich in the literature of the eighteenth and nineteenth centuries, and there are very few medical works of importance published within the last fifty years which are not to be found upon its shelves. Since the year 1800 about one-half of the bulk of medical literature consists of journals and transactions, and of these this library contains about 95 per cent. of those which have any value as works of reference, being 29,017 volumes. The number of current medical journals now received is 728, besides 43 pharmaceutical, 37 dental, and 58 popular medicine journals, and 130 miscellaneous scientific periodicals, which contain more or less matter of medical interest. These figures do not include transactions of societies, annual reports of hospitals, etc., of which the files are kept as complete as possible.

If one wishes to search for reports of cases or of operations, or for published medical statistical material with regard to diseases or injuries, he will find more in this library than in any other single collection in the world. If, however, he seeks data for local medical history, or for publications of the 15th or 16th centuries the case is different. The formation of this library may be said to date from about 25 years ago, at which time it contained two or three thousand volumes. It has been fairly successful in obtaining the older literature and there are very few ancient medical authors of repute of whose published works it does not contain at least one edition, but in number and completeness of editions, and of rare pamphlets, it is surpassed by several of the large old libraries of Europe, and notably by the British Museum and the Bibliotheque National of Paris. In the Repertorium Bibliographicum of Hain, which was intended to give an account of all books printed prior to the year 1500 A.D., there are noted about 800 editions of medical works. It is now, however, known that of works exclusively medical, there were about 1,200 different editions of about 430 distinct books printed prior to 1500. Of these, the so-called medical incunabulae, the Washington Library, has only about 100, or, say, one tenth of the whole, so that this is not the place in which one may properly expect to find many of these curiosities, specimens of which are scattered through the great National and University Libraries of Europe.

So also, if one is seeking documents relating to the history of English or French Medical Corporations or Societies, or hospitals or asylums, it is to the London and Paris collections that he will naturally first turn, although we are bound to say that he will often be disappointed.

The Washington Library is well housed in a plain fire-proof building, which also contains the Army Medical Museum, and which is large enough to contain all the additions which are likely to be made to it for at least ten years to come. But this library is a long way off from Europe, and is not even situated in one of the great medical centres of the United States, so that if its formation had only consisted in bringing the books together and placing them on shelves it would have been of little interest to the medical profession at large, or even in its own country.

That which has made this library really important and useful is its catalogue, copies of which are accessible in every large city in Europe. This catalogue is one of both Authors and subjects arranged in alphabetical order, and under each subject is given, not only the titles of the books and pamphlets but also the titles of the principal articles, or papers in journals, or transactions, pertaining to it with the proper references. This makes it more of an index to the contents of the library than catalogues usually are, and hence it is called "The Index Catalogue." Up to the present time eleven volumes of this work have been printed, including the alphabet from "A" to "Regent," and probably about four more volumes will be required to complete this series of the work. The published parts give under names of authors 117,327 titles, including 58,833 volumes, and 101,910 pamphlets; and under names of subjects, 121,681 book titles, and references to 374,852 journal articles. One volume is published each year—it is therefore eleven years since the first volume was published—and the additions during that time to the collection of literature, which in the alphabetical classification of authors and subjects belongs to volume one is very large. By the time the volume containing Z is published there will probably have accumulated a manuscript catalogue of supplementary titles sufficient to make four or five printed volumes of a new series.

From one point of view it is a little discouraging to reflect that such a work as this can never be made complete and up to date in print for general use, although it is of course constantly kept complete in manuscript, and it is very easy to be pessimistic about the steadily increasing bulk of medical literature, and the very transient value and interest which most of it possesses. It would be a bad sign, however, if the increase were to cease, and we may safely leave to future bibliographers and investigators to deal with the half million or so medical books which may be in existence a century hence.

The utility of the Index Catalogue to European physicians is, of course, chiefly bibliographical, to give them a starting point in their researches by enabling them to note certain books, and especially certain journals, which they will desire to see and which they can find in libraries in their vicinity or possibly even on their own shelves. It is a work which requires a little study as to its arrangement and system of cross references to enable one to use it to the best advantage, but any one who is hunting up the literature of the subject will find that he can get some help from it. The most exasperating thing about it in its present incomplete condition is that one is apt to want information about subjects which come in the latter part of the alphabet and are therefore not yet printed, but this defect is steadily lessening each year.

To keep the collection complete in such matters as reports of hospitals, medical and nurses' training schools, health offices, &c., is more difficult than to obtain the latest text books and monographs, and the librarian is constantly writing for documents of this kind. We commend his requests to the favourable consideration of those of our readers who may receive them. We know of no other library which makes a systematic effort to collect and preserve such things from all parts of the world, and such effort is worthy of aid and encouragement.

